# Media use during adolescence: the recommendations of the Italian Pediatric Society

**DOI:** 10.1186/s13052-019-0725-8

**Published:** 2019-11-27

**Authors:** Elena Bozzola, Giulia Spina, Margherita Ruggiero, Davide Vecchio, Cinthia Caruso, Mauro Bozzola, Anna Maria Staiano, Rino Agostiniani, Antonello Del Vecchio, Giuseppe Banderali, Diego Peroni, Alberto Chiara, Luigi Memo, Renato Turra, Giovanni Corsello, Alberto Villani

**Affiliations:** 1 Italian Pediatric Society, Rome, Italy; 2 University of Tor Vergata, Rome, Italy

**Keywords:** Media device, Adolescence, Smartphone, Addiction, Internet

## Abstract

**Background:**

The use of media device, such as smartphone and tablet, is currently increasing, especially among the youngest. Adolescents spend more and more time with their smartphones consulting social media, mainly Facebook, Instagram and Twitter because. Adolescents often feel the necessity to use a media device as a means to construct a social identity and express themselves. For some children, smartphone ownership starts even sooner as young as 7 yrs, according to internet safety experts.

**Material and methods:**

We analyzed the evidence on media use and its consequences in adolescence.

**Results:**

In literature, smartphones and tablets use may negatively influences the psychophysical development of the adolescent, such as learning, sleep and sigh. Moreover, obesity, distraction, addiction, cyberbullism and Hikikomori phenomena are described in adolescents who use media device too frequently. The Italian Pediatric Society provide action-oriented recommendations for families and clinicians to avoid negative outcomes.

**Conclusions:**

Both parents and clinicians should be aware of the widespread phenomenon of media device use among adolescents and try to avoid psychophysical consequences on the youngest.

## Background

Media device use, especially interactive apps, including social network and video games, is severely increasing in childhood [1].

Considering social network, Facebook is the most used platform with 2.4 billion users worldwide followed by Instagram and Twitter [2].

In particular, among adolescents, the age of initial use of social network is dropping to 12–13 years nowadays because of the necessity to use it as a means to construct a social identity and express themselves [2] [3].

According to ISTAT, 85.8% of italian adolescents aged 11–17 years have regular access to smartphones, and over 72% access Internet via smartphones. More girls (85.7%) use smartphone compared to boys [4]. Moreover, recent studies reported that 76% of adolescents use social network, with 71% of them using more than one social network apps [5]. Almost half of the adolescents are constantly online [6].

Online communication, education and entertainment are increasingly taking place online. In Europe, Eurostat analysis evidenced a great growth of Internet access from 55% in 2007 to 86% in 2018, and Internet access through a mobile device from 36% in 2012 to 59% in 2016 [7, 8].

Considering worldwide data, the number of smartphone users is forecast to reach 2.87 billion users in 2020 [9].

Moreover, problematic internet use is actually considered an important public health concern in specific groups such as the adolescents. For example, Chinese and Japan studies report that 7.9 to 12.2% of adolescents were problematic Internet users [10, 11]. In India, the prevalence is even higher, reaching 21% in vulnerable groups [12].

In Italy few data exist about media use in adolescence [4, 13, 14].

A survey pointed out that 75% of adolescents use a smartphone during school activities and 98% use it over midnight. Many adolescents sleep with their smartphone under pillows (45%) and check smartphone during the night (60%). Moreover, 57% of them use smartphone within ten minutes from awaking and 80% fall asleep holding their smartphone [14].

### Aim

Aim of the study is to describe the evidence on media use and its consequences among adolescents.

## Materials and methods

For the purpose of the study we investigated both positive and negative outcomes of media use on adolescents, considering health related problems, in order to give recommendations to optimize the use and to reduce negative consequences. A search strategy consisting of a systematic review of the thematic scientific literature published from January 2000 to April 2019 using the Preferred Reporting Items for Systematic Reviews and Meta-Analyses (PRISMA) guidelines. A comprehensive literature search of the MEDLINE/PubMed, Cochrane Library, the Cumulative Index to Nursing and Allied Health Literature (CINHAL) databases was conducted. The search algorithm was based on a combination of the following terms: media use, social network, video games, childhood, adolescence, family, parents, smartphone, internet, learning, sleep, sight, addiction, muscle, distraction, hikikomori, social withdrawal, cyberbullying, positive aspects, negative aspects. No language restriction was applied.

## Results

### Learning

Social network and smartphone may be related to learning consequences, such as low academic outcomes, reduced concentration and procrastination [15–17].

Problematic smartphone use (PSU) correlates to a surface approach to learning more than to a deep approach one [18]. Among the negative consequences of a surface approach, the most frequent are: reduced creativity, organization skills, own thinking and comprehension of information [19, 20]. Moreover, students with a surface approach to learning just aim to do only what is strictly necessary to study, reaching less satisfactory results than deeper learners [15, 21–24].

### Sleep

According to a recent literature review, the use of media devices during bed-time is frequent: 72% of children and 89% of adolescents have at least one media device in the bedroom [25]. The pre-sleep smartphone use has been reported to interfere with both sleep duration and quality [26, 27].

Moreover, a lot of health problems have been described in relation to poor sleep quality: alcohol use disorders, depression, eye syndromes, body fatigue, obsessive-compulsive disorder and increased susceptibility to colds and fever [28–33].

The circadian rhythm can be negative influenced by pre-sleep smartphone use, leading to inadequate sleeping: increased sleep latency, arousal and reduced sleep duration by approximately 6.5 h on weekdays [34–36].

Electromagnetic radiations and bright smartphones lights can cause physical discomfort such as muscular pain or headaches [37–39].

In addition, recent researches suggested that either inadequate sleep quality or sleep duration are related to metabolic conditions such as diabetes and cardiovascular disease or psychological problems such as depression or substance abuse [40, 41].

The number of adolescents with a sleep duration shorter than the one recommended by National sleep Foundation duration has increased, mainly among the girls (45.5% vs 39.6% in boys) [42].

Finally, a 5 or more hours daily of media devices use has been related to a higher risk of sleep problems when compared to a 1 h use daily [43].

### Sight

The increased use of smartphones may result in ocular problems such as dry eye disease (DED), eye irritation and fatigue, burning sensation, conjunctival injection, decreased visual acuity, strain, fatigue acute acquired comitant esotropia (AACE) and macular degeneration [44, 45].

During smartphone use there is a reduction of the blink rate to 5–6/min that promotes tear evaporation and accommodation, leading to DED [46–48]. Luckily, a 4 week stop of smartphone use may led to clinical improvement in DED patients [49].

As for AACE, the close reading distance can increase the medial rectus muscles tone, causing an alteration of both vergence and accommodation. As well as in DED, clinical symptoms may improve refraining from smartphones [50, 51].

### Addiction

One of the most problematic aspects of smartphones and Internet use in adolescents is addiction. Addiction is referred to someone obsessed by a specific activity which interferes with dailies activities [52].

In case of smartphone addiction, persons continuously check e-mails and social apps. The easy access to smartphone skills during the day facilitates the spreading of this kind of addiction [53]. The smartphone use even during a face-to-face communication is an increased phenomena as well. It is called “phubbing” [54].

As suggested by previous studies, smartphone addiction may be compared to substance-use addiction [55].

Diagnostic criteria for smartphone addiction have been proposed in order to facilitate an early recognition of it [56].

According to the Teen Smartphone Addiction National Survey questionnaire conducted from 2016 to 2018, 60% of teens’ friends, in their estimation, are addicted to their phones [57]. Actually, few countries classify addiction as a disease. This is probably the reason why we have few data on media device addiction in adolescence.

A recent Survey by the National Information Society Agency in 2012 evidenced that smartphone addiction in Chorea was 8.4% [58].

Some Studies emphasized risk factors related to smartphones addiction such as personality and sociodemographic features but also parental attitude. In details, concern, loss of controlling tolerance, withdrawal, instability and impulsiveness, mood modification, lies, loss of interest have been identified as risk factors of smartphone addiction [59].

Considering gender factors, previous researches described that females spent more time on smartphones and had almost 3 times more risk for smartphone addiction than males [60, 61]. It has also been reported that female addiction may be related to a stronger desire for social relationships [62].

Regarding parental attitude toward smartphone use, parental education is important to treat adolescents with addictions [63, 64]. In this context, parents may prevent smartphone addiction among adolescents by providing support. In fact, a good parent–adolescent relationship may reduce social anxiety and increase security and self-esteem [65]. On the other hand, parental attachment and insecurity may increase the risk of smartphones addiction in adolescents [66].

Main psychological problems correlated to addiction are: low self-esteem, stress, anxiety, depression, insecurity and solitude [18, 67].

School outcomes may be affected as well because smartphone addiction may led adolescents to ignore responsibilities and to spend time unproductively [68, 69].

Internet is often used to escape from negative feelings and solitude, avoid face-to-face interactions, increase self-esteem, enhancing the risk of depression, social anxiety and addiction [70, 71].

Smartphone addiction has been related to two phenomena: fear of missing out (FOMO) and boredom.

FOMO may be described as the apprehension of loose experiences and the consequent wish to remain constantly socially connected with the others. FOMO generates the necessity to check continuously social app in order to keep up to date on friends’ activities [72].

Boredom is defined as an unpleasant emotional state, related to lack of psychological involvement and interest associated with dissatisfaction. People may try to cope with boredom by seeking additional stimulation and compulsively using smartphones [73–75].

Adolescents, who are more vulnerable, have a higher risk of boredom and of pathological use of on-line communication applications [76]. On the contrary, smartphone addiction could be negatively influenced by face to face adolescent contacts [77].

### Muscle and skeleton

Problematic smartphone use (PSU) has been related to skeletal problems, muscles pain, sedentary lifestyle, lack of physical energy and weakened immunity [78, 79].

Some Chinese reports, describe that 70% of adolescents experienced neck pain, 65% shoulder pain, 46% wrist and finger pain. Musculoskeletal disorders related to smartphones may be influenced by many factors, including smartphone display size, number of text messages sent and hours daily spent on smartphones [80, 81].

Moreover, during smartphone use, a non physiological posture may lead to cervical problems. For example, a neck flexion (33–45°) may cause musculoskeletal consequences especially in the neck region [82, 83].

In particular, texting is one of the most contributing factor of stress on the cervical spine and of neck pain in those who spent 5.4 h a day on their smartphone [82, 84].

### Distraction

Smartphones activities are associated with higher cognitive distraction and with lower awareness occasionally endangering the lives of users [85].

The risk of distraction is higher in case of large smartphone screens and in case of gaming [86].

Dramatic data showed that vehicle crashes are one of the major causes of injuries in children. The USA experienced an increase of 5% of motor vehicle fatalities in adolescents [87, 88]. This may be related to a PSU. In fact, pedestrians using internet and smartphones have a high risk to engage traffic accident because they less frequently look both way and cross the road with a minor attention [89]. In particular, music listeners have a decreased situational awareness [90].

In this context, the role of parental modeling is crucial in adolescent behaviors development: adolescents with parents engaging in cell phone-related distracted driving are more likely to use a cell phone while driving themselves. A study conducted on 760 parents while children (4–10 years) were in the vehicle observed that 47% of parents talked on a hand-held phone, 52.2% talked on a hands-free phone, 33.7% read text messages, 26.7% sent text messages, and 13.7% used social network while driving [91]. This could be a very dangerous and continuously increasing phenomenon involving adolescents and future adults.

### Cyberbullying

The increasing rate of cyberbullying is related to a wide availability of smartphones, internet and mobile devices. It may be defined as a form of bullying performed by a person or by a group through an electronic mean and finalized to inflict discomfort, threat, fear or embarassment to the victim [92]. There are different forms of cyberbullying described by literature: phone calls, text messages, pictures/video clips, emails and messaging apps are among the most used [93]. This is a great public health concern: in Italy, 2015 ISTAT data showed that 19.8% of 11–17 years old internet users, report being cyberbullied [94–96].

### Hikikomori

A social phenomenon called *Shakaiteki hikikomori* (social withdrawal) is becoming increasingly recognized in several Countries [97]. To date, it has been estimated that approximately 1–2% of adolescents and young adults are hikikomori in Asian countries. Most of them are males and experience a social reclusion ranges from 1 to 4 years [98–104]. They refuse to communicate even with their own family, continuously use Internet and only venture out to deal with their bodily needs.

Many hikikomori spends even more than 12 h a day in front of a screen and consequently is at high risk of internet addiction [105–107].

### Positive aspects

Smartphone and Internet have also been related to numerous positive aspects concerning with social interactions and communication, developmental and psychology features.

Adolescents may improve self-control, express opinions and reflective decisions [108].

Adolescents who feel isolated and depressed, may establish relationships without concerning about how others evaluate their physical aspect, improve their depressed mood and gain support to increase their self-esteem and peer acceptance and obtain emotional support [109–113].

Results are summarized in Table 1.

**Table 1:** Main reviewed articles and their principal features

DOMAIN	REFERENCE	TYPE OF STUDY DESIGN	METHOD	MAIN INSTRUMENTS USED	OUTCOMES’ ANALISYS AND/OR STANDARDIZED GRADING OF EVIDENCE LEVEL APPLIED	HIGHLIGHTED PROS/CONS
Learning	Rogaten J et al., 2013 [15]	CS	Online survey	Approaches and Study Skills Inventory for Students, the Positive and Negative Affect Schedule, the Evaluation Anxiety Scales	SA	Low academic outcomes, reduction of concentration, reduction of creativity, reduction of comprehension, reduction of organization
	Kirschner PA et al., 2016 [16]	CS	Customized survey	Five sections of closed-response (e.g., Likert-type scaling) and open-response items.	SA	
	Lopez-Fernandez O et al., 2017 [18]	CS	Online survey	The dependence subscale of a short version of the Problematic Mobile Phone Use Questionnaire	SA	
	Arquero JL, at al., 2015 [22]	CS	Customized survey	New Study Process Questionnaire (N-SPQ-3f) and Academic Motivation Scale	Statistical and comparative subgroups’ analysis of the observed data	
	Rozgonjuk D et al., 2018 [24]	CS	Online survey	Estonian Smartphone Addiction Proneness Scale and the Estonian Revised Study Process Questionnaire	SA	
Sleep	Charter B et al., 2016 [25]	MA	RW	24 MeSH terms, titles and abstracts were screened for relevance	Statistical heterogeneity was assessed using the I2 statistic	Increase of sleep latency, increase of arousals, reduction of sleep duration, metabolic issues
	Prather AA et al., 2014 [31]	CS	Longitudinal study	NA	SA	
	Caine N et al., 2010 [37]	DS	RW	Literature Research	SA	
	Bixler E, 2009 [41]	DS	RW	Literature Research	SA	
	Owens J, 2014 [42]	DS	RW	Literature Research	SA	
Sight	Fenga C et al., 2014 [47]	CC	NA	Ocular examination and questionnaire	SA	Dry eye disease, eye irritation and burning sensation, conjunctival injection, decrease visual acuity, acute acquired comitant esotropia, macular degeneration
	Moon JH et al., 2014 [48]	CC	NA	Ocular examination and questionnaire	SA	
	Moon JH et al., 2016 [49]	CC	NA	Ocular examination and questionnaire	SA	
Addiction	Chotpitayasunondh V et al., 2016 [54]	CS	Online survey	Phubbing questionnaire, Smartphone Addiction Scale Short Version (SAS-SV), Internet Addiction Test (IAT), Fear of Missing Out Scale (FoMOs), and Brief Self-Control Scale (BSCS)	Statistical analysis of the observed data	Fear of missing out, wish to remain constantly connected, necessity to check continuously social app, boredom, lack of psychological involvement, lack of interest dissatisfaction
	Wegmann E et al., 2016 [55]	CS	Survey	Modified Version of the Short Internet Addiction Test, internet-Use Expectancies Scale, Brief COPE, elf-Efficacy Scale, Brief Symptom Inventor,	Statistical analysis of the observed data	
	Choi SW et al., 2015 [60]	CS	Survey	Smartphone Addiction Scale, the Young Internet Addiction Test, the Alcohol Use Disorders Identification Test, the Beck Depression Inventory I, the State–Trait Anxiety Inventory (Trait Version), the Character Strengths Test, and the Connor-Davidson Resilience Scale	Multiple linear regression analyses of observed data	
	Long J et al., 2016 [62]	CS	Survey	Socio-demographic, smartphone use feature, problematic Cellular Phone Use Questionnaire (PCPUQ), Chinese Frost Multidimensional Perfectionism Scale (CFMPS), Zung Self-Rating Depression Scale (SDS), Zung Self-Rating Anxiety Scale (SAS), Perceived Stress Scale (PSS)	Statistical analysis of the observed data	
	Jia R et al., 2016 [65]	CHS	Anonymous survey	Measurement scales to assess PIU and parental attachment (both maternal and paternal)	Ordinary least squares regression	
	Liu M et al. 2016 [67]	MA	PRISMA guideline	Electronic databases of PubMed, Web of Science and EBSCO systematically review	Generalised least squares trend estimation	
Muscle	Lee JH et al., 2014 [81]	CC	Survey	Questionnaires	SA	Neck pain, shoulder pain, wrist and finger pain, vehicle crashes, traffic accident
	Kang JH et al., 2012 [83]	CC	Survey	Computer software	SA	
	Collet C et al.,2010 [87]	DS	Survey	Questionnaires	SA	
Distraction	Collet C et al.,2010 [87]	DS	Survey	Questionnaires	SA	Vehicle crashes, traffic accidents
	Stelling-Konczak A et al., 2017 [90]	DS	Survey	Questionnaire	SA	
	Byington KW et al., 2013 [91]	CS	Survey	Virtual pedestrian street Questionnaire	SA	
	Bingham CR et al., 2015 [93]	DS	Survey	National sample of 403 parent-teen dyads using random-digit dialing telephone interviews.	Bingham CR et al., 2015 [93]	
Cyberbullying	Tokunaga RS, 2010 [94]	RW	Survey	Questionnaires	SA	Social discomfort, exclusion, alienation,
	Smith PK et al., 2008 [95]	DS	Survey	Questionnaires	SA	
Hikikomori	Stip E et al., 2016 [105]	DS	RW	Medline up to 12th May, 2015 supplemented by a hand search of the bibliographies of all retrieved articles.	SA	Absence of human-human interaction and communication
	Lee YS et al., 2013 [106]	CC	Interview	Participants’ Depression Inventory, Trait Anxiety Inventory, Social Anxiety Scale, and Internet Addiction Scale scores.	SA	
	Li TM et al., 2015 [107]	DS	RW	ProQuest, ScienceDirect, Web of Science and PubMed, quantitative and qualitative studies of socially withdrawn youths published in English and academic peer-reviewed journals.	NA	
Positive aspects	Ferrara P et al., 2014 [109]	DS	Customized internet-based	Newspaper indexes, news websites and internet search engines such as Google were used.	DS	Improvement of self-control, improvement of communication among depressed children, improvement of communication among insecure adolescents, improvement of capacity to express opinions
	Petry NM et al., 2014 [110]	RW	Experts’ estimations	Cross-cultural experts’ collaboration	NA	
	Ferrara P et al., 2018 [111]	DS	RW	NA	NA	
	Baer S et al., 2011 [112]	CS	Survey	Computer/Gaming-station Addiction Scale	SA	

Legend as follow: NA, not available feature; CC, case control; DS, descriptive study; CHS, cohort study; RP, report; RW, review; MA, metanalysis; SA, statistical analysis

## Discussion

### Advices

#### To parents

On the base of literature reports, parents should be aware of positive and negative effects of smartphone and media device use in adolescents. Consequently, action-oriented recommendations for families include:

- Improve communication: invite adolescents to critically discuss about the time they spent on media device and about the social app they use. Encourage them sharing problems they may experience offline and online. Aware them on online content and on online privacy.
- Monitor: verify the time spent online and the contents; promote active discussion about media device use; suggest co-view and co-play.
- Define clear policies and regulations**:** avoid media device use during meals, homeworks and bed-time.
- Give the example: reduce time spent using smartphones during family meeting, when crossing the street and during meals.
- Collaboration: create a network with pediatricians and health care providers in order to aware adolescent internet and smartphones disorders.

#### To clinicians

On the base of literature reports, recommendations for clinicians and health care providers include:

- Communication with adolescents and parents: inform adolescents on positive and on negative effects of media device use. Provide information on: addiction risk, distraction, academic outcomes, neuropsychological consequences, comprehension. Discuss with adolescents about their smartphones and social network use, approaching it in a more conscious and informed way. Reflect with teens and parents about how screen-based distractions around are linked with impaired academic performance and how parents are an important model for their children.
- Social networks and positive aspects: discourage adolescents use of social network and smartphones just to avoid solitude and to increase self-esteem; promote a safe use of media to connect with friends and share contents.
- Improve student–student relationship: promote face-to face relationship with adolescents and family.
- Recognize changes in health and social behaviour: in order to promptly copy with smartphone addiction and to minimize negative effects, clinicians should recognize symptoms and signs suggestive for a not correct media device use, such as weight gain/loss, headaches and muscle pain, vision/eye disturbances, etc.
- Educate: introduce screening questions about child’s on-line life into the general pediatric visit, including questions about video game use and cyberbullying, in order to identify adolescents that are engaging in health risk behaviours or addiction problems.
  Advices are summarized in Table 2.

**Table 2:** Advices to parents and clinicians on media use during adolescence

ADVICES			
ITEM	TO PARENTS	ITEM	TO CLINICIANS
Communication	- Create open communication sharing problems and difficulties - Remind the need to protect online privacy - Encourage critical thinking about media	**Communication**	- Provide to adolescents Information about positive and negative effects of media use - Reflect with teens about worsening of academic performances
Monitoring	- Time spent online - The appropriate technology for each stage of development. - Contents	**Positive aspects**	- Promote face-to face interaction - Encourage a safe use of media - Promote media use to share contents with friends
Rules	- No media use during meals - No media use during home-works - No media use during bed time	**Physical examination and education**	- Introduce screening questions about media use during general visit - Recognize symptoms and signs suggestive for a not correct media use: weight loss/gain headaches, muscle discomfort, eyes disturbances, psychological problems
Give the example	- Restrict time spent using smartphone during family meetings - Do not use smartphones when driving and during meals. - Choose appropriate contents for online communication and social network	**Collaboration**	- Create a network with families and health care providers
Collaboration	- Create a network with pediatrician and health care providers		

## Conclusion

Smartphones and social network have become integral part of adolescent’s life influencing the person’s whole life. Both parents and clinicians/ health care providers should understand both potential benefits and risks in order to prevent negative consequences, such as smartphone addiction. Both clinicians and parents should strive to better understand adolescent online activities, discuss with them about smartphone use and prevent adverse events.
